# A novel pilot animal model for bone augmentation using osseous shell technique for preclinical in vivo studies

**DOI:** 10.1002/cre2.644

**Published:** 2022-08-07

**Authors:** Mohammad Kamal, Sara Al‐Obaidly, Bernd Lethaus, Alexander K. Bartella

**Affiliations:** ^1^ Department of Surgical Sciences, Faculty of Dentistry, Health Sciences Center Kuwait University Jabryia Kuwait; ^2^ Kuwait Dental Administration Kuwait Ministry of Health Safat Kuwait; ^3^ Department of Oral and Maxillofacial Surgery Leipzig University Hospital Leipzig Germany

**Keywords:** animal model, bone augmentation, bone grafting, osteogenesis

## Abstract

**Objectives:**

Bone grafting is commonly used to reconstruct skeletal defects in the craniofacial region. Several bone augmentation models have been developed to evaluate bone formation using novel bone substitute materials. The aim of this study was to evaluate a surgical animal model for establishing a three‐dimensional (3D) grafting environment in the animal's mandibular ramus for bone augmentation using the osseous shell technique, as in humans.

**Materials and Methods:**

Osteological survey of New Zealand white (NZW) rabbit skull (*Oryctolagus cuniculus*): Initial osteological and imaging surveys were performed on a postmortem skull for a feasibility assessment of the surgical procedure. Postmortem pilot surgery and cone beam computed tomography imaging: a 3D osseous defect was created in the mandibular ramus through a submandibular incision. The osseous shell plates were stabilized with osteosynthesis fixation screws, and defects were filled with particular bone grafting material. In vivo surgical procedure: surgeries were conducted in four 8‐week‐old NZW rabbits utilizing two osseous shell materials: xenogeneic human cortical plates and autogenous rabbit cortical plates. The created 3D defects were filled using xenograft and allograft bone grafting materials. The healed defects were evaluated for bone formation after 12 weeks using histological and cone beam computed tomography imaging analysis.

**Results:**

Clinical analysis 12 weeks after surgery revealed the stability of the 3D grafted bone augmentation defects using the osseous shell technique. Imaging and histological analyses confirmed the effectiveness of this model in assessing bone formation.

**Conclusions:**

The proposed animal model is a promising model with the potential to study various bone grafting materials for augmentation in the mandibular ramus using the osseous shell technique without compromising the health of the animal. The filled defects could be analyzed for osteogenesis, quantification of bone formation, and healing potential using histomorphometric analysis, in addition to 3D morphologic evaluation using radiation imaging.

## 1 INTRODUCTION

Bone is a dynamic tissue made of organic and inorganic components with a unique healing capacity that allows it to generate new cells without scarring. Bone formation occurs through endochondral or intramembranous ossification (Kamal et al., 2018; Ozaki & Buchman, 1998). Endochondral bone formation begins with the formation of a cartilage template followed by gradual ossification, and it occurs mostly in growth plates and in long bones, such as the tibia, femur, and radius. On the other hand, intramembranous bone formation starts with the formation of clusters of dense mesenchymal stem cells, which then differentiate into osteoblasts and directly produce osteoid, for example, in the skull and ribs. Smaller bone defects can heal spontaneously without intervention. Larger bone defects, however, do not spontaneously heal and require interventional procedures to enhance bone generation in a process called bone grafting or an augmentation procedure.

Bone grafting is a common surgical method used in craniofacial surgery to fix skeletal defects, fill bone gaps, and improve the physiological healing of wounds. (Gordh, 1999; Nyström et al., 1996) The current gold standard is the autogenous bone transplant, although its usage is limited due to many factors, including surgical morbidity, limited bone supply in donor sites, intrinsic graft resorption, uncertain graft volume stability, and the need for a second surgical operation for harvesting the bone transplant, which leads to increased time and resources required (Gordh, 1999; Gordh et al., 1998; Lappalainen, Karhula, Haapea, Kyllönen, et al., 2016; Nyström et al., 1996, 2002, 2004; Saikia et al., 2008). In 2007, Khoury developed a novel approach for grafting alveolar ridge defects (Khoury & Hanser, 2015, 2019). This procedure entailed harvesting thin cortical plates from the ramus and interposing those bone plates with cancellous bone taken from the same place in a “sandwich” style (Khoury & Hanser, 2015, 2019). Consequently, this approach remains to have the same drawbacks as conventional harvesting approaches. Considering recent technological advances in biomedical research, the biological alternative to conventional bone grafting is the utilization of a variety of renewable resources for bone replacement. Several resorbable and non‐resorbable bone substitute materials have been utilized as suitable replacement materials for craniofacial reconstruction, including processed allografts (Clokie et al., 2002; Haddad et al., 2006), xenografts (Su‐Gwan et al., 2001), alloplasts such as bioactive ceramics like hydroxyapatite and tricalcium phosphate (Clokie et al., 2002; Lappalainen, Karhula, Haapea, Kauppinen, et al., 2016; Saikia et al., 2008; Zhou et al., 2007), and polymer‐based materials like polyether ether ketone and fiber‐reinforced bioglass (Lappalainen, Karhula, Haapea, Kyllönen, et al., 2016; Lappalainen, Karhula, Haapea, Kauppinen, et al., 2016). Peck reported the use of allograft bone plates instead of harvesting cortical bone plates to reconstruct lost alveolar bone in three dimensions (Peck, 2015).

Interventional testing of novel bone grafting substitute materials in vivo is necessary, as it allows for the application of realistic modeling to evaluate functional recovery in a biologic environment and to determine clinical osteogenesis efficacy (Kamal, Andersson, Tolba, Al‐Asfour, et al., 2017). The regenerative capacity and osteogenesis sequence of a novel material for bones have been intensively investigated using in vivo animal models (Haddad et al., 2006; Humber et al., 2010; Lappalainen, Karhula, Haapea, Kyllönen, et al., 2016; Lappalainen, Karhula, Haapea, Kauppinen, et al., 2016). The rabbit calvarium originates embryonically out of a membrane precursor, replicating the intramembranous ossification of the facial bones, making it a better paradigm/model for translational evaluation in craniofacial studies (Clokie et al., 2002; Hussain et al., 2012; Schmitz & Hollinger, 1986). However, this model was not able to assess the vertical bone regeneration capacity of the grafting material tested. This is not reflective of the clinical setting, since most defects have some vertical elements that require bone regeneration in the vertical dimension. Hence, recent studies have attempted to establish a rabbit model for the evaluation of the vertical bone regeneration capacities of various biomaterials. Various bone augmentation models have been built to improve the notion of “guided bone regeneration (GBR)” in the oral and craniofacial surgical domains. The essential idea behind this concept is that newer bone tissue is formed vertically on top of the cortical region of the skull. This has been previously described and achieved using several vertical containers (e.g., dome/cylinder/cage made of titanium, polyether ether ketone (PEEK), poly‐l‐lactic acid (PLLA), ceramic) that were fixed onto the skull to protect and contain the bone regeneration conducted by a grafting material (Anderud et al., 2014; Chakar et al., 2015; Ikeno et al., 2013; Lee et al., 2003; Lima et al., 2018; Marger et al., 2019; Pieri et al., 2010; Polo et al., 2013; Sudheesh Kumar et al., 2018; Tamimi et al., 2006; Wang et al., 2015; Yamada et al., 2008).

The objective of this study was to evaluate a lab animal model in New Zealand white (NZW) rabbits (*Oryctolagus cuniculus)* as a grafting environment for bone augmentation utilizing the osseous shell approach, just as it is performed in humans.

## 2 MATERIALS AND METHODS

### 2.1 Osteological survey of NZW rabbit skull (*O. cuniculus*)

A skull of a deceased NZW rabbit from the Kuwait University Health Sciences Center Animal Resources House was obtained, and radiographic imaging was obtained using cone beam computed tomography (CBCT) for inspection and evaluation of anatomical structures (J. Morita Corp.). CBCT images were analyzed in multiple dimensions to assess the feasibility of using various anatomical areas to create an appropriate vertical and horizontal bone defect model. The ramus of the NZW rabbit mandible showed a uniform concavity that could be accessed via a surgical submandibular approach to expose the ramus for surgery. Further review and assessment of the complete anatomical landmarks of the skull were discussed with a specialized veterinarian with expertise in conducting surgeries on rabbits. Cross‐sectional slices and three‐dimensionally produced reconstruction pictures were used to evaluate the images.

### 2.2 Postmortem pilot surgery and CBCT imaging

**Figure 1:**
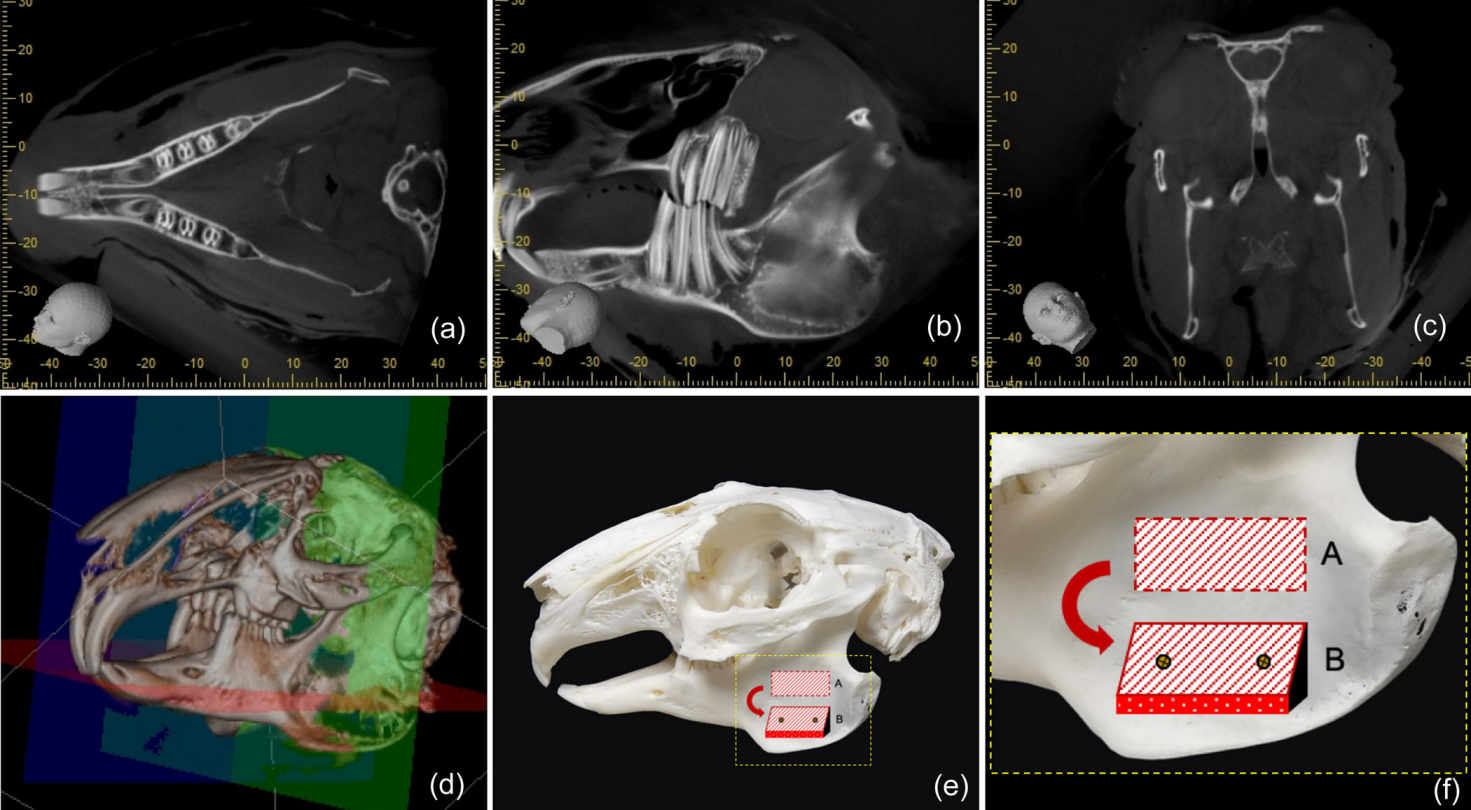
Osteological survey of a preserved New Zealand white rabbit mandible (*Oryctolagus cuniculus*). (a–d) The ramus shows an anatomical area and morphology suitable for creating a three‐dimensional osseous shell model. (e and f) Here, the osseous shell was drawn to be taken for the superior part of the ramus and inferiorly fixed at more inferior to create the shell model.

**Figure 2:**
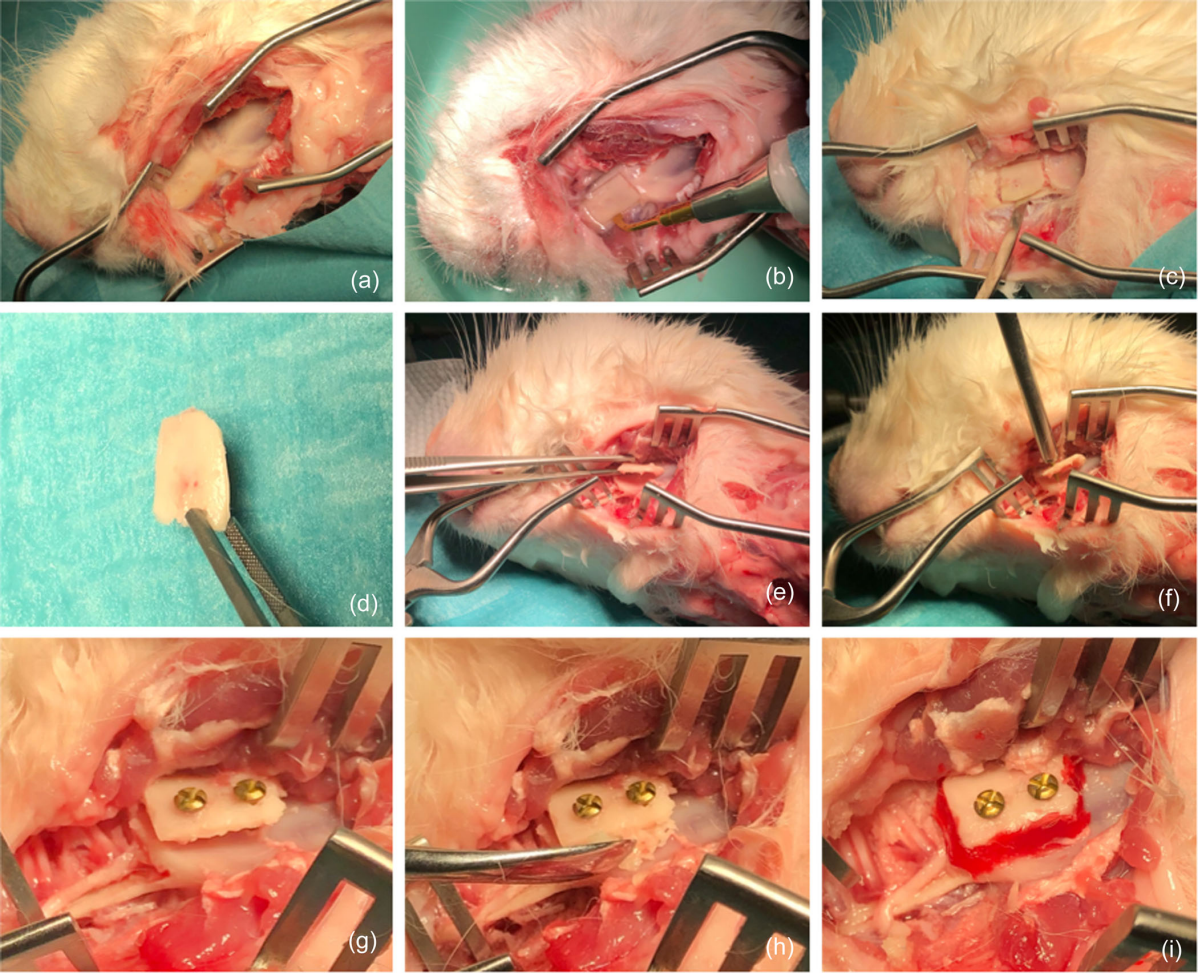
(a–e) Postmortem pilot surgery on a killed NZW rabbit showing the surgical access to reach the mandible and harvest the cortical bone shelf. The defect can be properly manipulated, fixated, and grafted and then completely covered by primary closure of the skin to allow healing and create a closed biological environment. (f–i) Fixation of the osseous shell and filling the defect with grafting material.

In a pilot study on a dead rabbit's head, the proposed technique was employed to ensure its applicability. Considering that the surgery was conducted on a dead animal that had already been utilized in another animal research experiment, no clearance from an animal ethics council was required (Figure 1). The three‐dimensional mandibular bone augmentation model surgery was conducted on the ramus of the mandible according to the shell technique, ascertaining the feasibility of this procedure on live rabbits to create a horizontal and vertical defect model (Figure 2).

### 2.3 In vivo animals, anesthesia, and housing

This study was approved by the Animal Ethics Committee of the Kuwait University Health Sciences Center, Kuwait (3755/2021). The live animal surgery was carried out at Kuwait University's Animal Research Center, Health Sciences Center (HSC) according to our previously utilized protocols (Kamal, Andersson, Tolba, Bartella, et al., 2017, 2018). All animals were handled in compliance with the European Communities Council Directive of November 24, 1986 (86/609/EEC) for the management of lab animals in experimental procedures as well as ethical principles for animal research and the ARRIVE guidelines (Directive, 1986). All procedures were designed to comply with the Health Sciences Center's policies and standards after approval from the HSC Central Ethical Committee for Use of Laboratory Animals in Teaching and Research at Kuwait University. The experiment included four 8‐week‐old NZW male rabbits weighing 2.7–3.0 kg. The rabbits were anesthetized 30 min before the experimental procedure with a xylazine HCl intramuscular injection (Rompun) at a dose of 5 mg/kg and then sedated with ketamine HCl at a dose of 35 mg/kg via intravenous injection (Tekan). A veterinarian administered the drugs and anesthesia to ensure that all animal care procedures were followed while sedating the animals and that other care‐taking practices were observed throughout the study (Al‐Asfour et al., 2013; Andersson et al., 2009).

### 2.4 Preparation of surgical sites and in vivo surgical procedure

**Figure 3:**
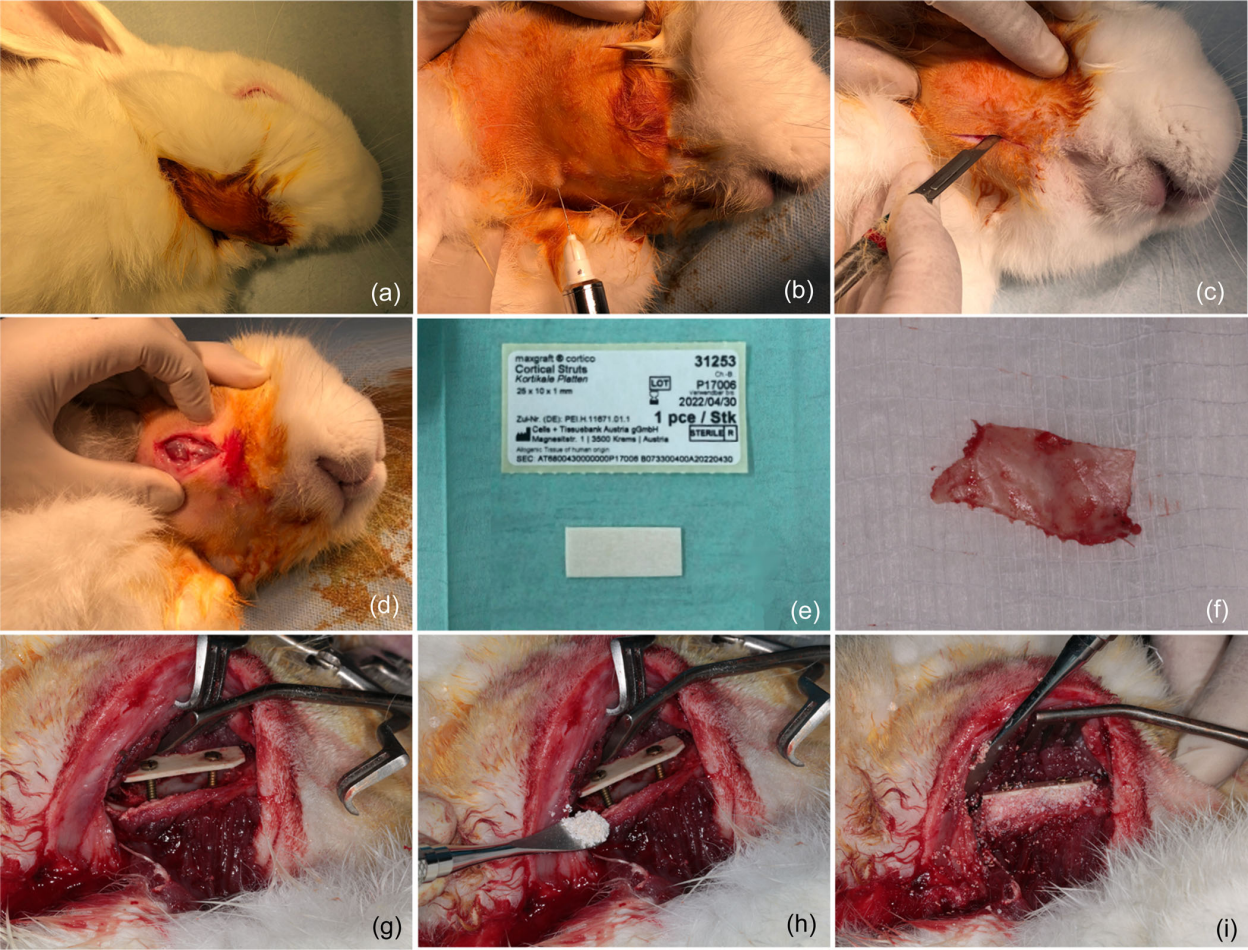
(a–i) In vivo osseous shell model creation surgery. Surgical access was achieved through the submandibular approach after prepping, draping, and application of local anesthesia with the preparation of the tissue to reach the mandible. Osseous shells could be created with a xenogeneic (human) cortical sheet (Maxgraft Cortico, Botiss Biomaterials GmbH, Zossen), or autogenous bone harvested from the superior ramus. The cortical shell was fixed with 1.2 mm osteosynthesis screws (USTOMED INSTRUMENTE, Ulrich Storz GmbH & Co., Tuttingen). The three‐dimensional defect was filled with xenogeneic (bovine) bone substitutes (Bio‐Oss, Geistlich Pharma AG) and cerabone (Botiss Biomaterials GmbH).

Once the rabbit was sedated, a lubricating eye gel was applied to the rabbit's eyes to avoid dryness and irritation. The surgical site was then shaved and disinfected with povidone 10% iodine gel. The animals were draped with a sterile surgical drape with a cut‐out window to access the surgical site. Local anesthesia was then administered (Lidocaine 2%, 1 ml). A linear incision was made in the submandibular area, along with the rabbit's mandibles. To reveal the bone defect in the jaw, the periosteum was cut vertically with a scalpel and gently reflected using a periosteal elevator. Once the concavity of the ramus was reached, the soft tissue was further prepared and lifted in the lateral and superior directions to allow full exposure to the site. To utilize a proper osseous shell to create the three‐dimensional defect, two of the most commonly utilized bone shells in clinical practice were used in this animal study: autogenous bone shell and xenograft bone shell. Autogenous bone was harvested from the superior part of the ramus in the rabbits using a piezoelectric surgery device (Mectron Piezosurgery) (Figure 3).

In rabbit no. 1, cerabone (Botiss Biomaterials GmbH, Zossen, Germany) was used with a 2.0 × 10 mm processed human cortical bone plate (Maxgraft Cortico, Botiss Biomaterials GmbH), which was stabilized with two 1.2 × 11 mm screws (USTOMED Instrumente, Ulrich Storz GmbH & Co.) and then secured with porcine pericardium membrane (Bioguide, Geistlich Pharma AG). For rabbit no. 2, an autogenous cortical plate was harvested as described. A bone scraper was used to retrieve some autogenous bone particles, which were then mixed with cerabone bovine bone. The autogenous cortical plate was then secured with two 11‐mm screws and then covered with a porcine pericardium membrane. In rabbit no. 3, a combination of cerabone and Bio‐Oss (Geistlich Pharma AG) was used with a 2.0 × 10 mm allograft cortical plate, which was also stabilized with two 1.2 × 11 mm screws and then secured with Bioguide membrane. To prevent thermal and mechanical damage to the bone and soft tissue, an autogenous cortical plate was resected in rabbit no. 4 using a piezoelectric surgical tool and copious irrigation with sterile saline. A bone scraper was used to retrieve some autogenous bone particles, which were then mixed with Bio‐Oss. The autogenous cortical plate was then secured with two 11‐mm screws and covered with a Bioguide membrane. Resorbable sutures (Vicryl®, Ethicon Inc.) were used to close the wounds in multiple layers. Throughout the trial, the animals were housed in separate containers and fed soft pellets and water. The rabbits were cared for following standard animal research procedures and were monitored by a full‐time veterinarian until the end of the experiment.

### 2.5 Animal sacrifice and qualitative evaluation

Following anesthesia with an intramuscular injection of xylazine HCl at a dose of 5 mg/kg, animals were killed 90 days after surgery with an intravenous fatal dosage of T61 Euthanasia Solution (Embutramide, Mebezonium iodide, Tetracaine hydrochloride; Hoechst GmbH).

### 2.6 Postoperative CBCT of the filled defects

After the animals were killed, postoperative imaging of the four surgical specimens was obtained using CBCT. The images were obtained using high‐resolution CBCT at 70 kV, 4 mA, and 0.125 mm pixel size (J. Morita Corp.).

### 2.7 Histologic preparation, histomorphometry, and statistical analysis

After image acquisition, the specimens were submerged and fixed for 48 h in 10% neutral buffered formalin. The specimens were decalcified in neutral EDTA for 1 week, dehydrated in alcohol, and vacuum‐embedded in paraffin using standard histological techniques. Serial sections were cut at a thickness of 5 μm, mounted on polylysine‐coated slides, stained with hematoxylin and eosin, and examined using light microscopy. The best sections, comprising both cortex and marrow areas, were selected and evaluated using light microscopy (Leitz) at a magnification of 1.25x. Sections were evaluated with regard to tissue morphology, signs of inflammation, and the formation of new bone.

## 3 RESULTS

### 3.1 Postmortem pilot surgery

The simulation of the three‐dimensional bone augmentation testing model on a postmortem rabbit skull using the osseous shell approach indicated the feasibility of establishing a sufficiently large defect, replicating what is specified for use in humans. The defect could be easily accessed via the surgical submandibular approach, with an adequate access area for visualization and surgical manipulation during the procedure.

### 3.2 In vivo surgery in NZ white rabbits

The entire process took 25–35 min for each rabbit. All procedures were performed under ketamine anesthesia, with appropriate pain management provided by local anesthetic infiltration, without the need for endotracheal intubation. A second injection of ketamine was necessary for a few subjects. Minor bleeding occurred during and after the surgical operation, but no significant arterial bleeding was observed throughout the surgery. The animals were active, acted in a normal way during the healing period, and immediately began eating shortly following surgery. Throughout the experiment, the animals were fed ad libitum. All of the rabbits survived the 12‐week postoperative period until the day of sacrifice.

### 3.3 CBCT imaging of healed defects postsurgery

**Figure 4:**
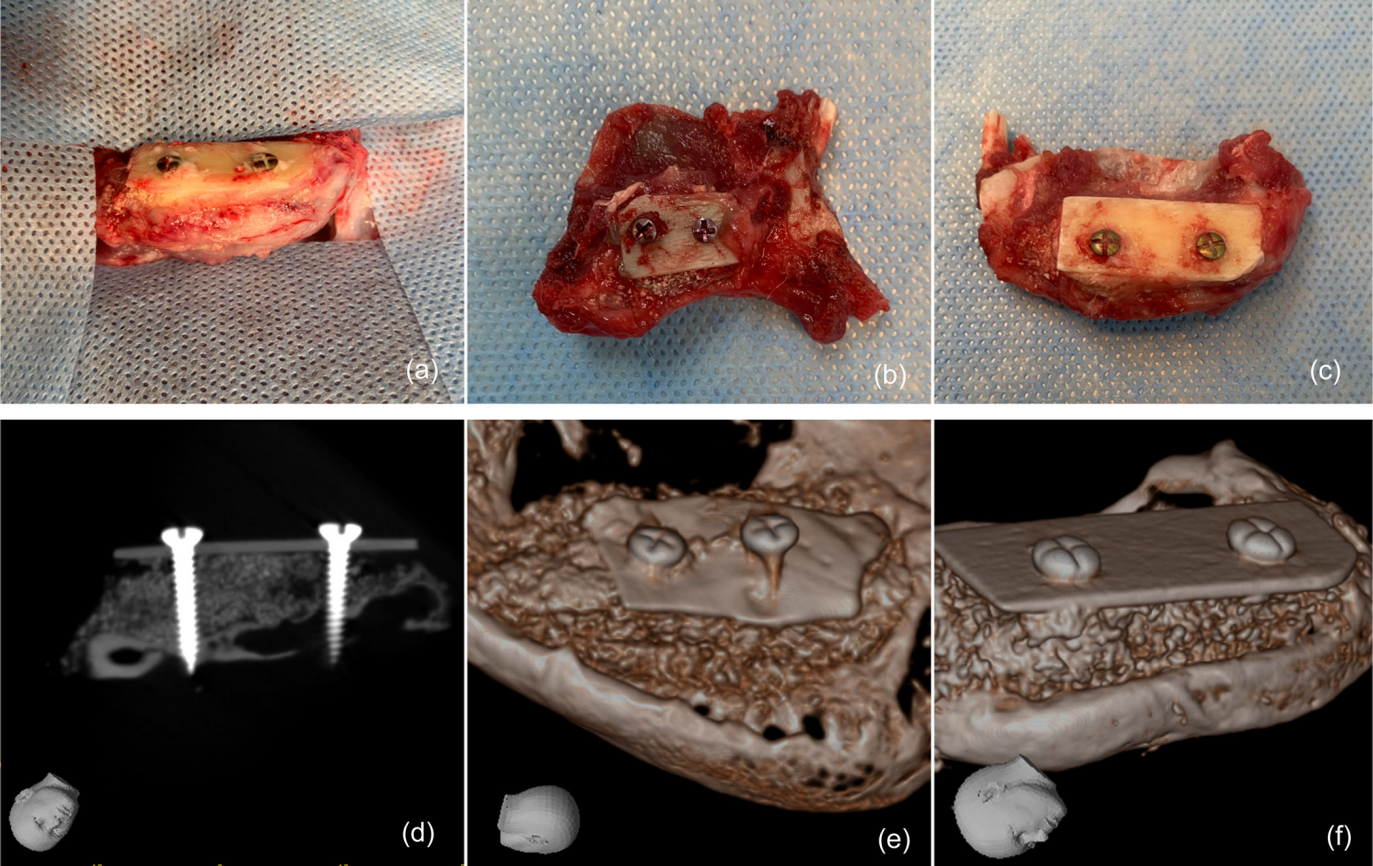
(a–c) Postoperative specimens and cone beam computed tomography (CBCT) images. (d) Block sections of the mandible containing the grafted shell specimens. (d–f) CBCT imaging reconstruction of the grafted osseous shell defect at 12 weeks after in vivo surgery (J. Morita Corp.). (e) Shell created using autogenous bone. (f) Shell created using xenogeneic (human) cortical bone.

After 12 weeks of healing and eventual animal sacrifice, CBCT images were obtained for the four surgical specimens (J. Morita Corp.). 3D imaging reconstruction of the defect revealed a properly sized augmented bone defect that simulated the conventional osseous shell technique for bone augmentation in humans (Figure 4). The images were of good resolution and displayed the exact shape of the augmented defect, localization of the placed screws, and the integrated bone substitute materials used in the grafting intervention.

### 3.4 Histological assessment of healed defects

**Figure 5:**
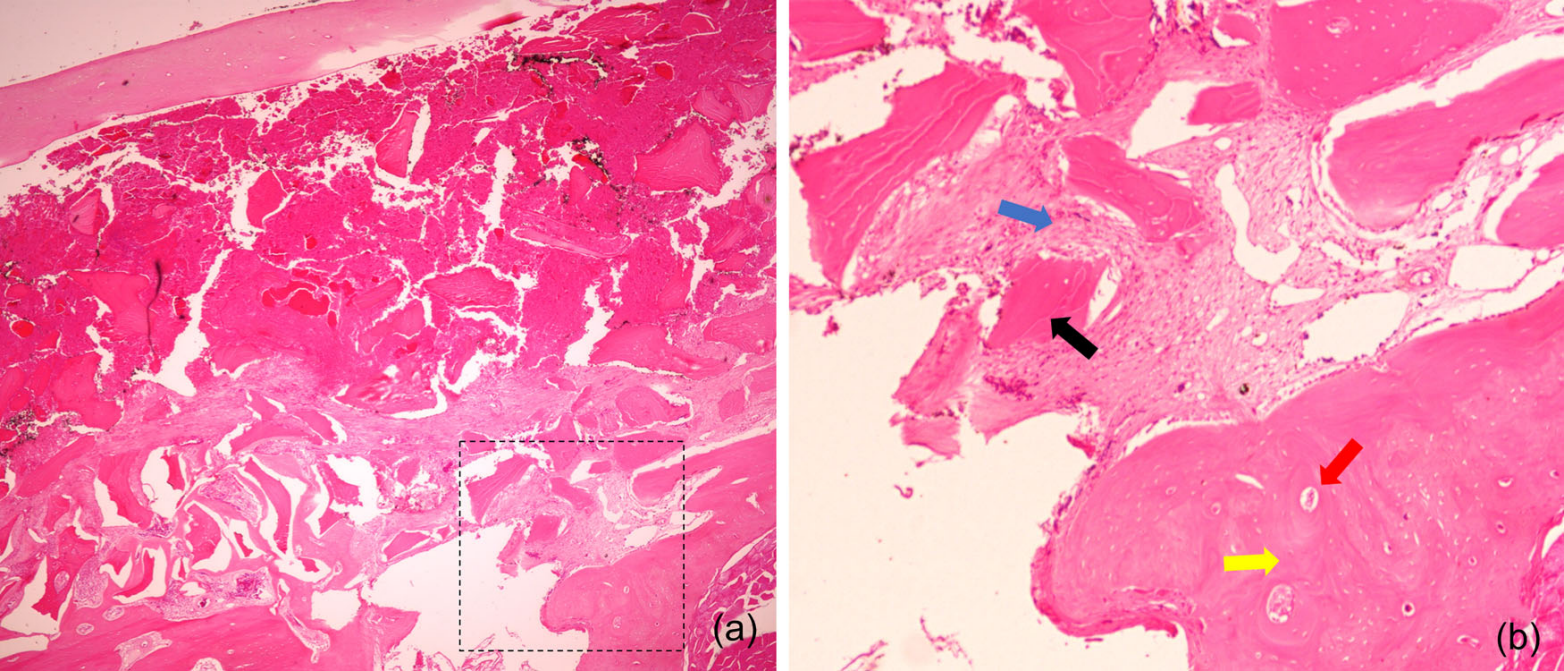
Histological section of the grafted osseous shell stained with Hematoxylin and Eosin 12 weeks after surgery demonstrating residual grafting materials surrounded by newly formed bone, fused grafting particles with bone, and cortical shell with discrete lacunae. (a) Low magnification 4x. (b) High magnification 40x. Black arrow: residual particulate bone graft; blue arrow: newly formed bone; red arrow: osteocytic lacunae; yellow arrow: grafted cortical shell.

Histological analysis after 12 weeks demonstrated a bone healing pattern in the osseous shell and particulate bone grafts, which is in accordance with what was seen in the CBCT images. Islands of osteocytes and new bone formations can be seen in addition to resorption lacunae, which are associated with bone substitute materials undergoing osseous formation and bone remodeling. The screws were fully integrated and entirely encompassed by newly generated bone tissue, as seen in the slides. Preserved collagen membrane structures were observed on the surface of the generated alveolar ridge. The pins showed little resorption and were mostly embedded in firm tissue. Besides the pinheads, periosteal new bone development that incorporated the pins into the bone tissue was visible. Representative histological images of tissue formation in distinct groups at 14 weeks are provided in Figure 5.

## 4 DISCUSSION

The aim of this study was to develop a laboratory animal model in NZW rabbits that could be used to establish a three‐dimensional grafting environment for bone augmentation using the osseous shell technique, as done in human patients. Laboratory animals commonly used in biomaterial bone studies include small animals, such as mice, rats, hamsters, guinea pigs, and rabbits, as well as large animals, such as goats, dogs, and primates (Li et al., 2015; Mapara et al., 2012; Pearce et al., 2007). Rodent models have inherent limitations when compared to larger animal models. Rodents have smaller long bones and more fragile cortices, and they do not show Haversian‐type remodeling in the cortex (Li et al., 2015). Rabbits are the largest species in the small animal category, so they are least susceptible to the complex and stringent extra clearance processes that central ethics committees normally impose. They are nonaggressive, easy to examine, have a faster vital ability to mature and breed, and can be locally bred (Kamal, Andersson, Tolba, Al‐Asfour, et al., 2017; Li et al., 2015; Mapara et al., 2012; Pearce et al., 2007). The histology of rabbit bone differs from that of humans: it consists of thick Haversian bone and layers of vascular‐longitudinal canals (Li et al., 2015; Mapara et al., 2012; Pearce et al., 2007). Nonetheless, similarities in human and rabbit bone mineral density and hardness have been shown in previous publications (Mapara et al., 2012; Pearce et al., 2007; Wang et al., 1998). Rabbits, in contrast to rodents, have accelerated skeletal metabolism and enhanced bone turnover (primarily cortical remodeling) (Li et al., 2015; Mapara et al., 2012; Pearce et al., 2007). The rabbit model is useful for bone augmentation experiments because the animal is reproducible, is convenient to house and anesthetize, provides large surgical fields for conducting technical procedures, and can withstand the trauma of surgery (Mapara et al., 2012; Pearce et al., 2007).

Autogenous bone grafts are often regarded as the gold standard in augmentative surgical procedures, particularly vertical augmentations (De Stavola et al., 2017; Gordh 1999; Nyström et al., 2002; Saikia et al., 2008; Tunkel et al., 2021). The major issues reported in the literature on autogenous full‐block transplants include resorption rates ranging from 21% to 25%, as well as limited supply (Khoury & Hanser, 2015; Nkenke & Neukam, 2014; Tunkel et al., 2021). Khoury describes the shell technique to avoid these issues (Khoury & Hanser, 2015, 2019; Tunkel et al., 2021). The shell approach makes effective use of bone block, allowing for considerably bigger bone volume to be supplemented with the same harvesting volume, while at the same time, resorption rates are decreased to 5%–9% by adhering to the basics of wound healing principles. (De Stavola & Tunkel, 2013a, 2013b; Tunkel et al., 2021). Vertical bone augmentations and the shell approach, in particular, are advanced technical procedures that require advanced surgical skills and technical preparation to obtain the desired results (De Stavola & Tunkel, 2013a, 2013b; Draenert et al., 2014, 2017; Tunkel et al., 2021; Xiao et al., 2019).

In modern clinical practice, many bone grafting materials and membrane techniques, with or without bone morphogenetic proteins, transforming growth factors, platelet‐derived growth factors, and basic fibroblast growth factors, have also been reported to regenerate bone efficiently (Gellrich et al., 2007). With the current use of human allogeneic bone cortices, further intraoral or extraoral bone block harvesting can indeed be foregone, minimizing the total risk of problems and patient morbidity, while providing clinical outcomes equivalent to those of autogenous bone shells (Stimmelmayr et al., 2012; Tunkel et al., 2021; Würdinger & Donkiewicz, 2020). Several clinical reports have indicated favorable outcomes with human allogeneic bone blocks for bone grafting procedures conducted according to the osseous shell technique (Tunkel et al., 2021; Würdinger & Donkiewicz, 2020). There is little evidence on the use of xenogeneic and allogeneic particulate bone material and blocks in various forms; there is even less literature on the use of allogeneic cortical plates for the shell approach. In initially disclosed case reports, the integration of human allogeneic bone plates and autogenous bone chips seems to be a viable substitute for autogenous implants in complex bone augmentation procedures. That being said, these studies were often case‐controlled cohorts, with no proper comparative studies published regarding credible animal models for in‐depth preclinical evaluation (Peck, 2015; Tunkel et al., 2021; Würdinger & Donkiewicz, 2020). Several studies have reported reductions in bone resorption rate and modulation of bone turnover and formation by using barrier membranes over augmented bone blocks (Antoun et al., 2001; De Stavola & Tunkel, 2013a; Iglhaut et al., 2014). These adjunctive interventions prompt us more toward developing a reliable animal model that provides a solid biological basis for further in vivo testing of bone grafting techniques to optimize clinical outcomes (De Stavola et al., 2017).

Our proposed model presents several advantages with regard to previous studies, in which the animals are large enough to bear the trauma of the surgery and provide the surgeon with a sizable bone augmentation working model in the mandibular ramus of rabbits. The size and topography of the defect in our model mimic the size and the working environment in humans and in the field of oral implantology and bone grafting. In addition, the defect can easily be reached and created with minimal blood loss, which makes the animals able to continue their oral intake and physical activity without much compromise to their health, making them useful for studies requiring a longer period of healing after surgery to assess osteogenic outcome. The bone augmentation model in ramus was never described before in the literature, and we propose that it would be a suitable and safe environment for the testing of the osseous shell technique as regulated three‐dimensional bone augmentation, as seen by the excellent clinical handling of the material and the ease of approach.

Limitations of the in vivo model are mainly related to the anatomy of the ramus in NZW rabbits, which could be anatomically atrophic in some animals requiring proper fixation by multiple osteosynthesis screws. Moreover, the size of the defect depends on the utilized bone shell, whether it is autogenous or xenograft, which prompts thorough planning by the operator to achieve a standardized defect size, especially with regard to the spacing between native bone and the grafted shell. This would possibly require the surgeon to utilize prefabricated fixation guides and blocks to achieve proper standardization of defect size. Nonetheless, the clinical applicability of the model may be more challenging in complicated defect patterns, necessitating more research.

## 5 CONCLUSIONS

The proposed animal model is a promising model with the potential to study various bone grafting materials for augmentation using the osseous shell technique. The model utilizes a sizable bone augmentation working model in the mandibular ramus of rabbits without compromising the health of the animal. The augmented defects with various bone‐substitute materials could be analyzed for osteogenesis, quantification of bone formation, inflammation, and healing potential using histomorphometric analysis, in addition to 3D morphologic evaluation using radiation imaging.

## AUTHOR CONTRIBUTIONS

Mohammad Kamal, Alexander K. Bartella, and Bernd Lethaus conceived and designed the study model. Mohammad Kamal, Sara Al‐Obaidly, and Alexander K. Bartella performed the postmortem procedure and defined the protocol. Mohammad Kamal and Sara Al‐Obaidly were responsible for the in vivo surgery and for performing the procedure. Mohammad Kamal, Sara Al‐Obaidly, Alexander K. Bartella, and Bernd Lethaus were responsible for image acquisition, histology, and analysis. Mohammad Kamal, Sara Al‐Obaidly, Alexander K. Bartella, and Bernd Lethaus have drafted the work or substantively revised it. All authors read and approved the final manuscript.

## CONFLICT OF INTEREST

The authors declare no conflict of interest.

## ETHICS STATEMENT

This study was approved by the Animal Ethics Committee of the Kuwait University Health Sciences Center, Kuwait (3755/2021). All animals were handled in compliance with the European Communities Council Directive of 24 November 1986 (86/609/EEC) for the care of laboratory animals in experimental procedures and ethical guidelines for animal research (Directive, 1986). All methods were carried out in accordance with the approved guidelines and regulations of the HSC Ethical Committee for the Use of Laboratory Animals in Teaching and in Research, Kuwait University.

## Data Availability

The data/material analyzed during the current study is available from the corresponding author upon request.

## References

[cre2644-bib-0001] Al‐Asfour, A. , Andersson, L. , Kamal, M. , & Joseph, B. (2013). New bone formation around xenogenic dentin grafts to rabbit tibia marrow. Dental Traumatology, 29(6), 455–460. 10.1111/edt.12045 23621118

[cre2644-bib-0002] Andersson, L. , Ramzi, A. , & Joseph, B. (2009). Studies on dentin grafts to bone defects in rabbit tibia and mandible; Development of an experimental model. Dental Traumatology, 25(1), 78–83. 10.1111/j.1600-9657.2008.00703.x 19208015

[cre2644-bib-0003] Anderud, J. , Jimbo, R. , Abrahamsson, P. , Isaksson, S. G. , Adolfsson, E. , Malmström, J. , Kozai, Y. , Hallmer, F. , & Wennerberg, A. (2014). Guided bone augmentation using a ceramic space‐maintaining device. Oral Surgery, Oral Medicine, Oral Pathology, and Oral Radiology, 118(5), 532–538. 10.1016/j.oooo.2014.06.011 25224903

[cre2644-bib-0004] Antoun, H. , Sitbon, J. M. , Martinez, H. , & Missika, P. (2001). A prospective randomized study comparing two techniques of bone augmentation: Onlay graft alone or associated with a membrane. Clinical Oral Implants Research, 12(6), 632–639. 11737108 10.1034/j.1600-0501.2001.120612.x

[cre2644-bib-0005] Chakar, C. , Soffer, E. , Cohen, N. , Petite, H. , Naaman, N. , & Anagnostou, F. (2015). Vertical bone regeneration with deproteinised bovine bone mineral or biphasic calcium phosphate in the rabbit calvarium: Effect of autologous platelet lysate. Journal of Materials Science. Materials in Medicine, 26(1), 5339. 10.1007/s10856-014-5339-5 25578693

[cre2644-bib-0006] Clokie, C. M. , Moghadam, H. , Jackson, M. T. , & Sandor, G. K. (2002). Closure of critical sized defects with allogenic and alloplastic bone substitutes. Journal of Craniofacial Surgery, 13(1), 111–121. 11887007 10.1097/00001665-200201000-00026

[cre2644-bib-0007] De Stavola, L. , Fincato, A. , Bressan, E. , & Gobbato, L. (2017). Results of computer‐guided bone block harvesting from the mandible: A case series. The International Journal of Periodontics and Restorative Dentistry, 37(1), e111–e119. 10.11607/prd.2721 27977816

[cre2644-bib-0008] De Stavola, L. , & Tunkel, J. (2013a). A new approach to maintenance of regenerated autogenous bone volume: Delayed relining with xenograft and resorbable membrane. International Journal of Oral & Maxillofacial Implants, 28(4), 1062–1067. 23869364 10.11607/jomi.2726

[cre2644-bib-0009] De Stavola, L. , & Tunkel, J. (2013b). Results of vertical bone augmentation with autogenous bone block grafts and the tunnel technique: A clinical prospective study of 10 consecutively treated patients. International Journal of Periodontics & Restorative Dentistry, 33(5), 651– 659. 23998161 10.11607/prd.0932

[cre2644-bib-0010] Directive, C. (1986). 86/609/EEC of 24 November 1986 on the approximation of laws, regulations and administrative provisions of the member states regarding the protection of animals used for experimental and other scientific purposes. Official Journal of the European Communities, 29, L358.

[cre2644-bib-0011] Draenert, F. G. , Gebhart, F. , Mitov, G. , & Neff, A. (2017). Biomaterial shell bending with 3D‐printed templates in vertical and alveolar ridge augmentation: A technical note. Oral Surgery, Oral Medicine, Oral Pathology, and Oral Radiology, 123(6), 651–660. 10.1016/j.oooo.2016.12.011 28215503

[cre2644-bib-0012] Draenert, F. G. , Huetzen, D. , Neff, A. , & Mueller, W. E. (2014). Vertical bone augmentation procedures: Basics and techniques in dental implantology. Journal of Biomedical Materials Research. Part A, 102(5), 1605–1613. 10.1002/jbm.a.34812 23733418

[cre2644-bib-0013] Gellrich, N. C. , Held, U. , Schoen, R. , Pailing, T. , Schramm, A. , & Bormann, K. H. (2007). Alveolar zygomatic buttress: A new donor site for limited preimplant augmentation procedures. Journal of Oral and Maxillofacial Surgery, 65(2), 275–280. 10.1016/j.joms.2005.11.081 17236933

[cre2644-bib-0014] Gordh, M. , Alberius, P. , Johnell, O. , Lindberg, L. , & Linde, A. (1998). Osteopromotive membranes enhance onlay integration and maintenance in the adult rat skull. International Journal of Oral and Maxillofacial Surgery, 27(1), 67–73. 9506306 10.1016/s0901-5027(98)80102-1

[cre2644-bib-0015] Gordh, M. A. P. (1999). Some basic factors essential to autogeneic nonvascularized onlay bone grafting to the craniofacial skeleton. Scandinavian Journal of Plastic and Reconstructive Surgery and Hand Surgery, 33(2), 129–146. 10450569 10.1080/02844319950159370

[cre2644-bib-0016] Haddad, A. J. , Peel, S. A. , Clokie, C. M. , & Sándor, G. K. (2006). Closure of rabbit calvarial critical‐sized defects using protective composite allogeneic and alloplastic bone substitutes. Journal of Craniofacial Surgery, 17(5), 926–934. 17003622 10.1097/01.scs.0000230615.49270.d1

[cre2644-bib-0017] Humber, C. C. , Sándor, G. K. , Davis, J. M. , Peel, S. A. , Brkovic, B. M. , Kim, Y. D. , Holmes, H. I. , & Clokie, C. M. (2010). Bone healing with an in situ–formed bioresorbable polyethylene glycol hydrogel membrane in rabbit calvarial defects. Oral Surgery, Oral Medicine, Oral Pathology, Oral Radiology, and Endodontology, 109(3), 372–384. 10.1016/j.tripleo.2009.10.008 20060340

[cre2644-bib-0018] Hussain, I. , Moharamzadeh, K. , Brook, I. M. , José de Oliveira Neto, P. , & Salata, L. A. (2012). Evaluation of osteoconductive and osteogenic potential of a dentin‐based bone substitute using a calvarial defect model. International Journal of Dentistry, 2012, 396316. 22505899 10.1155/2012/396316 PMC3312261

[cre2644-bib-0019] Iglhaut, G. , Schwarz, F. , Gründel, M. , Mihatovic, I. , Becker, J. , & Schliephake, H. (2014). Shell technique using a rigid resorbable barrier system for localized alveolar ridge augmentation. Clinical Oral Implants Research, 25(2), e149–e154. 23278408 10.1111/clr.12078

[cre2644-bib-0020] Ikeno, M. , Hibi, H. , Kinoshita, K. , Hattori, H. , & Ueda, M. (2013). Effects of the permeability of shields with autologous bone grafts on bone augmentation. The International Journal of Oral & Maxillofacial Implants, 28(6), e386–e392. 10.11607/jomi.te19 24278940

[cre2644-bib-0021] Kamal, M. , Andersson, L. , Tolba, R. , Al‐Asfour, A. , Bartella, A. K. , Gremse, F. , Rosenhain, S. , Hölzle, F. , Kessler, P. , & Lethaus, B. (2017). Bone regeneration using composite non‐demineralized xenogenic dentin with beta‐tricalcium phosphate in experimental alveolar cleft repair in a rabbit model. Journal of Translational Medicine, 15(1), 1–13. 29274638 10.1186/s12967-017-1369-3 PMC5742260

[cre2644-bib-0022] Kamal, M. , Andersson, L. , Tolba, R. , Bartella, A. , Gremse, F. , Hölzle, F. , Kessler, P. , & Lethaus, B. (2017). A rabbit model for experimental alveolar cleft grafting. Journal of Translational Medicine, 15(1), 50. 10.1186/s12967-017-1155-2 28235419 PMC5326493

[cre2644-bib-0023] Kamal, M. , Gremse, F. , Rosenhain, S. , Bartella, A. K. , Hölzle, F. , Kessler, P. , & Lethaus, B. (2018). Comparison of bone grafts from various donor sites in human bone specimens. Journal of Craniofacial Surgery, 29(6), 1661–1665. 29762319 10.1097/SCS.0000000000004586

[cre2644-bib-0024] Khoury, F. , & Hanser, T. (2015). Mandibular bone block harvesting from the retromolar region: A 10‐year prospective clinical study. The International Journal of Oral & Maxillofacial Implants, 30(3), 688–697. 26009921 10.11607/jomi.4117

[cre2644-bib-0025] Khoury, F. , & Hanser, T. (2019). Three‐dimensional vertical alveolar ridge augmentation in the posterior maxilla: A 10‐year clinical study. The International Journal of Oral & Maxillofacial Implants, 34(2), 471–480. 30883623 10.11607/jomi.6869

[cre2644-bib-0026] Lappalainen, O.‐P. , Karhula, S. , Haapea, M. , Kyllönen, L. , Haimi, S. , Miettinen, S. , Saarakkala, S. , Korpi, J. , Ylikontiola, L. P. , Serlo, W. S. , & Sándor, G. K. (2016). Bone healing in rabbit calvarial critical‐sized defects filled with stem cells and growth factors combined with granular or solid scaffolds. Child's Nervous System, 32(4), 681–688. 10.1007/s00381-016-3017-2 26782995

[cre2644-bib-0027] Lappalainen, O.‐P. , Karhula, S. S. , Haapea, M. , Kauppinen, S. , Finnilä, M. , Saarakkala, S. , Serlo, W. , & Sándor, G. K. (2016). Micro‐CT analysis of bone healing in rabbit calvarial critical‐sized defects with solid bioactive glass, tricalcium phosphate granules or autogenous bone. Journal of Oral & Maxillofacial Research, 7(2), 4. 10.5037/jomr.2016.7204 PMC4970504 27489608

[cre2644-bib-0028] Lee, Y. M. , Nam, S. H. , Seol, Y. J. , Kim, T. I. , Lee, S. J. , Ku, Y. , Rhyu, I. C. , Chung, C. P. , Han, S. B. , & Choi, S. M. (2003). Enhanced bone augmentation by controlled release of recombinant human bone morphogenetic protein‐2 from bioabsorbable membranes. Journal of Periodontology, 74(6), 865–872. 10.1902/jop.2003.74.6.865 12886998

[cre2644-bib-0029] Li, Y. , Chen, S.‐K. , Li, L. , Qin, L. , Wang, X.‐L. , & Lai, Y.‐X. (2015). Bone defect animal models for testing efficacy of bone substitute biomaterials. Journal of Orthopaedic Translation, 3(3), 95–104. 30035046 10.1016/j.jot.2015.05.002 PMC5982383

[cre2644-bib-0030] Lima, J. L. O. , Sendyk, D. I. , Sendyk, W. R. , Polo, C. I. , Correa, L. , & Deboni, M. C. Z. (2018). Growth dynamic of allogeneic and autogenous bone grafts in a vertical model. Brazilian Dental Journal, 29(4), 325–334. 10.1590/0103-6440201801994 30462757

[cre2644-bib-0031] Mapara, M. , Thomas, B. S. , & Bhat, K. (2012). Rabbit as an animal model for experimental research. Dental Research Journal, 9(1), 111–118. 22363373 10.4103/1735-3327.92960 PMC3283968

[cre2644-bib-0032] Marger, L. , Barone, A. , Martinelli‐Kläy, C. P. , Schaub, L. , Strasding, M. , Mekki, M. , Sailer, I. , Scherrer, S. S. , & Durual, S. (2019). Calvarial model of bone augmentation in rabbit for assessment of bone growth and neovascularization in bone substitution materials. Journal of Visualized Experiments, 13(150), e59976. 10.3791/59976 31475980

[cre2644-bib-0033] Nkenke, E. , & Neukam, F. W. (2014). Autogenous bone harvesting and grafting in advanced jaw resorption: Morbidity, resorption and implant survival. European Journal of Oral Implantology, 7(Suppl 2), S203–S217. 24977256

[cre2644-bib-0034] Nyström, E. , Ahlqvist, J. , Gunne, J. , & Kahnberg, K.‐E. (2004). 10‐year follow‐up of onlay bone grafts and implants in severely resorbed maxillae. International Journal of Oral and Maxillofacial Surgery, 33(3), 258–262. 15287309 10.1006/ijom.2003.0512

[cre2644-bib-0035] Nyström, E. , Ahlqvist, J. , Kahnberg, K.‐E. , & Rosenquist, J. (1996). Autogenous onlay bone grafts fixed with screw implants for the treatment of severely resorbed maxillae: Radiographic evaluation of preoperative bone dimensions, postoperative bone loss, and changes in soft‐tissue profile. International Journal of Oral and Maxillofacial Surgery, 25(5), 351–359. 8961015 10.1016/s0901-5027(06)80029-9

[cre2644-bib-0036] Nyström, E. , Ahlqvist, J. , Legrell, P. E. , & Kahnberg, K.‐E. (2002). Bone graft remodelling and implant success rate in the treatment of the severely resorbed maxilla: A 5‐year longitudinal study. International Journal of Oral and Maxillofacial Surgery, 31(2), 158–164. 12102413 10.1054/ijom.2001.0197

[cre2644-bib-0037] Ozaki, W. , & Buchman, S. R. (1998). Volume maintenance of onlay bone grafts in the craniofacial skeleton: Micro‐architecture versus embryologic origin. Plastic and Reconstructive Surgery, 102(2), 291–299. 9703062 10.1097/00006534-199808000-00001

[cre2644-bib-0038] Pearce, A. , Richards, R. , Milz, S. , Schneider, E. , & Pearce, S. (2007). Animal models for implant biomaterial research in bone: A review. European Cells & Materials, 13(1), 1–10. 17334975 10.22203/ecm.v013a01

[cre2644-bib-0039] Peck, M. T. (2015). Alveolar ridge augmentation using the allograft bone shell technique. The Journal of Contemporary Dental Practice, 16(9), 768–773. 26522605 10.5005/jp-journals-10024-1755

[cre2644-bib-0040] Pieri, F. , Lucarelli, E. , Corinaldesi, G. , Aldini, N. N. , Fini, M. , Parrilli, A. , Dozza, B. , Donati, D. , & Marchetti, C. (2010). Dose‐dependent effect of adipose‐derived adult stem cells on vertical bone regeneration in rabbit calvarium. Biomaterials, 31(13), 3527–3535. 10.1016/j.biomaterials.2010.01.066 20170950

[cre2644-bib-0041] Polo, C. I. , Lima, J. L. , De Lucca, L. , Piacezzi, C. B. , Naclerio‐Homem Mda, G. , Arana‐Chavez, V. E. , & Sendyk, W. R. (2013). Effect of recombinant human bone morphogenetic protein 2 associated with a variety of bone substitutes on vertical guided bone regeneration in rabbit calvarium. Journal of Periodontology, 84(3), 360–370. 10.1902/jop.2012.110674 22524330

[cre2644-bib-0042] Saikia, K. , Bhattacharya, T. , Bhuyan, S. , Talukdar, D. , Saikia, S. , & Jitesh, P. (2008). Calcium phosphate ceramics as bone graft substitutes in filling bone tumor defects. Indian Journal of Orthopaedics, 42(2), 169–172. 19826522 10.4103/0019-5413.39588 PMC2759621

[cre2644-bib-0043] Schmitz, J. P. , & Hollinger, J. O. (1986). The critical size defect as an experimental model for craniomandibulofacial nonunions. Clinical Orthopaedics and Related Research, 205, 299–308. 3084153

[cre2644-bib-0044] Stimmelmayr, M. , Guth, J. F. , Schlee, M. , Gohring, T. N. , & Beuer, F. (2012). Use of a modified shell technique for three‐dimensional bone grafting: Description of a technique. Australian Dental Journal, 57(1), 93–97. 10.1111/j.1834-7819.2011.01646.x 22369565

[cre2644-bib-0045] Sudheesh Kumar, P. T. , Hashimi, S. , Saifzadeh, S. , Ivanovski, S. , & Vaquette, C. (2018). Additively manufactured biphasic construct loaded with BMP‐2 for vertical bone regeneration: A pilot study in rabbit. Materials Science and Engineering C Materials Biological Applications, 92, 554–564. 10.1016/j.msec.2018.06.071 30184782

[cre2644-bib-0046] Su‐Gwan, K. , Hak‐Kyun, K. , & Sung‐Chul, L. (2001). Combined implantation of particulate dentine, plaster of Paris, and a bone xenograft (Bio‐Oss®) for bone regeneration in rats. Journal of Cranio‐Maxillofacial Surgery, 29(5), 282–288. 11673923 10.1054/jcms.2001.0236

[cre2644-bib-0047] Tamimi, F. M. , Torres, J. , Tresguerres, I. , Clemente, C. , Lopez‐Cabarcos, E. , & Blanco, L. J. (2006). Bone augmentation in rabbit calvariae: Comparative study between Bio‐Oss and a novel beta‐TCP/DCPD granulate. Journal of Clinical Periodontology, 33(12), 922–928. 10.1111/j.1600-051X.2006.01004.x 17092243

[cre2644-bib-0048] Tunkel, J. , de Stavola, L. , & Kloss‐Brandstätter, A. (2021). Alveolar ridge augmentation using the shell technique with allogeneic and autogenous bone plates in a split‐mouth design—A retrospective case report from five patients. Clinical Case Reports, 9(2), 947–959. 33598278 10.1002/ccr3.3626 PMC7869406

[cre2644-bib-0049] Wang, X. , Mabrey, J. D. , & Agrawal, C. M. (1998). An interspecies comparison of bone fracture properties. Biomedical Materials and Engineering, 8, 1–10. 9713681

[cre2644-bib-0050] Wang, X. , Zakaria, O. , Madi, M. , Hao, J. , Chou, J. , & Kasugai, S. (2015). Vertical bone augmentation induced by ultrathin hydroxyapatite sputtered coated mini titanium implants in a rabbit calvaria model. Journal of Biomedical Materials Research. Part B, Applied Biomaterials, 103(8), 1700–1708. 10.1002/jbm.b.33347 25533173

[cre2644-bib-0051] Würdinger, R. , & Donkiewicz, P. (2020). Allogeneic cortical struts and bone granules for challenging alveolar reconstructions: An innovative approach toward an established technique. Journal of Esthetic and Restorative Dentistry, 32(8), 747–756. 32920939 10.1111/jerd.12639

[cre2644-bib-0052] Xiao, W. , Hu, C. , Chu, C. , & Man, Y. (2019). Autogenous dentin shell grafts versus bone shell grafts for alveolar ridge reconstruction: A novel technique with preliminary results of a prospective clinical study. The International Journal of Periodontics and Restorative Dentistry, 39(6), 885–893. 10.11607/prd.4344 31613951

[cre2644-bib-0053] Yamada, Y. , Tamura, T. , Hariu, K. , Asano, Y. , Sato, S. , & Ito, K. (2008). Angiogenesis in newly augmented bone observed in rabbit calvarium using a titanium cap. Clinical Oral Implants Research, 19(10), 1003–1009. 10.1111/j.1600-0501.2008.01554.x 18828816

[cre2644-bib-0054] Zhou, A. J.‐J. , Peel, S. A. , & Clokie, C. M. (2007). An evaluation of hydroxyapatite and biphasic calcium phosphate in combination with Pluronic F127 and BMP on bone repair. Journal of Craniofacial Surgery, 18(6), 1264–1275. 17993867 10.1097/scs.0b013e318158cb1a

